# Thrombosis of acute superior mesenteric artery in a patient with breast cancer receiving toremifene therapy: a case report and literature review

**DOI:** 10.1186/s12905-023-02855-6

**Published:** 2024-01-03

**Authors:** Ximei Wang, Runhe Zhou, Dezong Gao

**Affiliations:** Department of General Surgery, Cheeloo College of Medicine, Qilu Hospital (Qingdao), Shandong University, Qingdao, 266035 China

**Keywords:** Superior mesenteric artery thrombosis, Toremifen, Breast cancer

## Abstract

It is widely recognized that cancer itself is related to increased risk of thromembolism. Venous thromboembolism is relatively common in breast cancer patients, but arterial thrombosis, especially acute superior mesenteric artery thrombosis (SMAT) associated with chemotherapy or endocrinotherapy, rarely occurs in breast cancer patients. There were few reports about acute SMAT in cancer patients who underwent chemotherapy, but no reports of acute SMAT caused by endocrine-therapy. We reported a 54-year-old patient with acute SMAT during toremifene treatment after breast cancer surgery. She underwent 4 cycles chemotherapy of TC regimen, then accepted toremifen endocrinotherapy because of positive estrogen receptor. She suffered from acute SMAT after 2 months toremifen treatment. Therefore, we consider that this case of acute SMAT may be a rare adverse event of toremifen. In view of the high risk and rarity of acute SMAT caused by toremifene, we suggest that except for venous thrombosis, arterial thrombosis in special position (ATSP) should be kept in mind during use of toremifene. Once a thrombotic event occurs, toremifene should be stopped immediately.

## Introduction

Superior mesenteric artery thrombosis (SMAT) is one cause of acute abdomen, which is usually caused by obstruction of the blood supply to the intestinal wall. SMAT presents clinically acute onset, rapid progression, easily leads to ischemic intestinal necrosis. Due to the lack of specific clinical manifestations, diagnosis and treatment are often delayed, and the fatality rate can be as high as 70–90% [1]. Therefore early diagnosis and treatment is crucial to decrease the fatal rate of SMAT.

Endocrinetherapy is an important measures in patients with breast cancer. Toremifene or tamoxifen is appropriate to treat estrogen receptor positive patients, and no significant differences in DFS and OS were found between treatment with either toremifene or tamoxifen [2]. Toremifene, like tamoxifen, is a nonsteroidal triphenylethylene selective estrogen receptor modulator that binds to estrogen receptors. It is reported toremifene and tamoxifen were equally well tolerated in the treatment of breast cancer patients [3].

The adverse events of toremifene and tamoxifen mentioned in the instruction manual include mainly gynecologic, ocular events, and thromboembolism. Thromboembolic events include deep vein embolism, pulmonary embolism and arterial thrombosis. Cardiac- and cerebra-vascular thrombosis are relative more arterial accidents, but arterial thrombosis in special position (ATSP) is unusual, that is, embolic artery exclude cardiac- and cerebra-vascular thrombosis. Although the metabolic mechanism of toremifene was not very clear, as a chlorinated derivative of tamoxifen, toremifene may result in different metabolism and toxicity profile [3].

In our study, we reported a 54-year-old patient with acute SMAT during toremifene treatment after breast cancer surgery, and reviewed the relevant literature reports and summarized the experiences and lessons on ATSP during endocrine-therapy. We suggest that during the use of toremifene, not only the occurrence of venous thrombosis should be closely monitored, but also the risk of arterial thrombosis, especially ATSP should be pay attention.

## Case report

A 54-year-old female arrived at the emergency room with a sudden left abdominal severe pain with nausea and vomiting without inducement for 2 h. Physical examination showed tenderness and rebound tenderness in left abdomen, but no abdominal rigidity. Her gurgling sound disappeared, and shifting dullness didn’t be found in abdomen.

The patient’s previous medical history was breast cancer, and now take toremifene for endocrine-therapy. The patient underwent mastectomy and sentinel lymph node biopsy 6 months ago, then accepted 4 cycles chemotherapy of TC regimen (paclitaxel + cyclophosphamide). Oral toremifene endocrine therapy began two months ago. The patient denied smoking history, had no history of arrhythmia, diabetes, peripheral vascular diseases or previous thromboembolic events, and her laboratory tests including serum lipid, platelet count and coagulation function were normal.

**Fig. 1:**
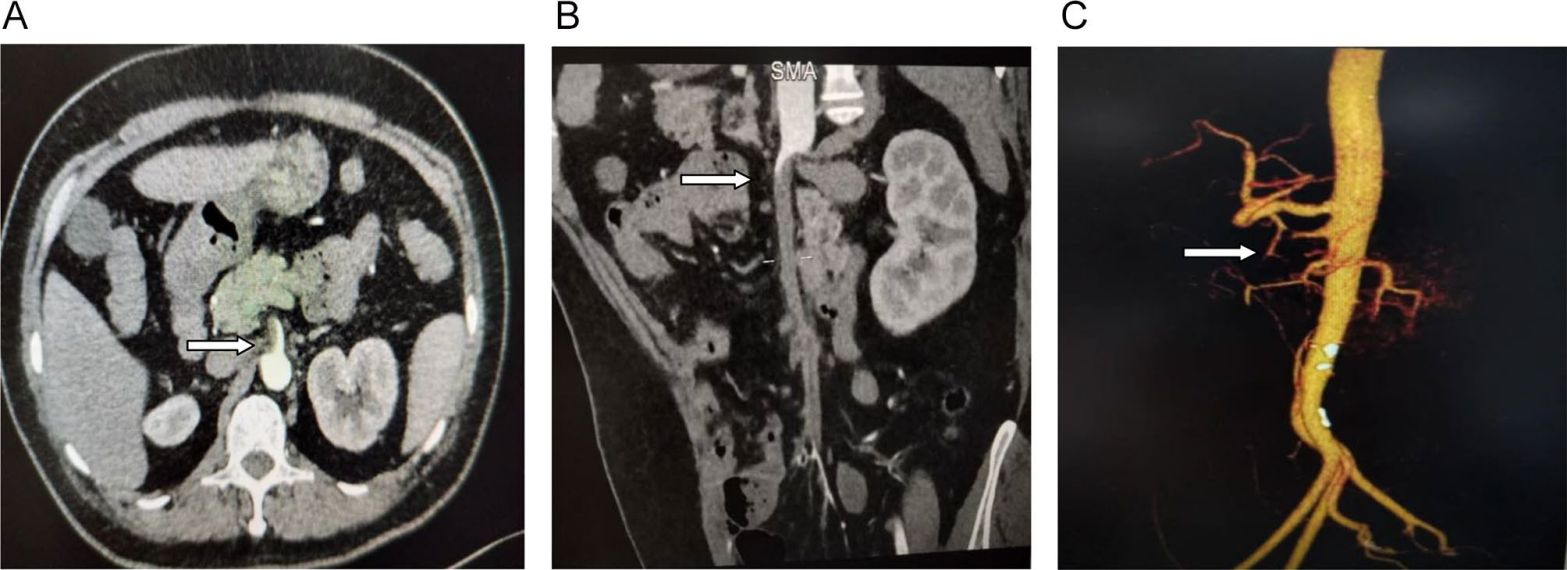
Superior mesenteric artery thrombosis in CTA (coronal (**A**) and sagittal (**B**) section). SMAT in three-dimensional reconstruction (**C**)

**Fig. 2:**
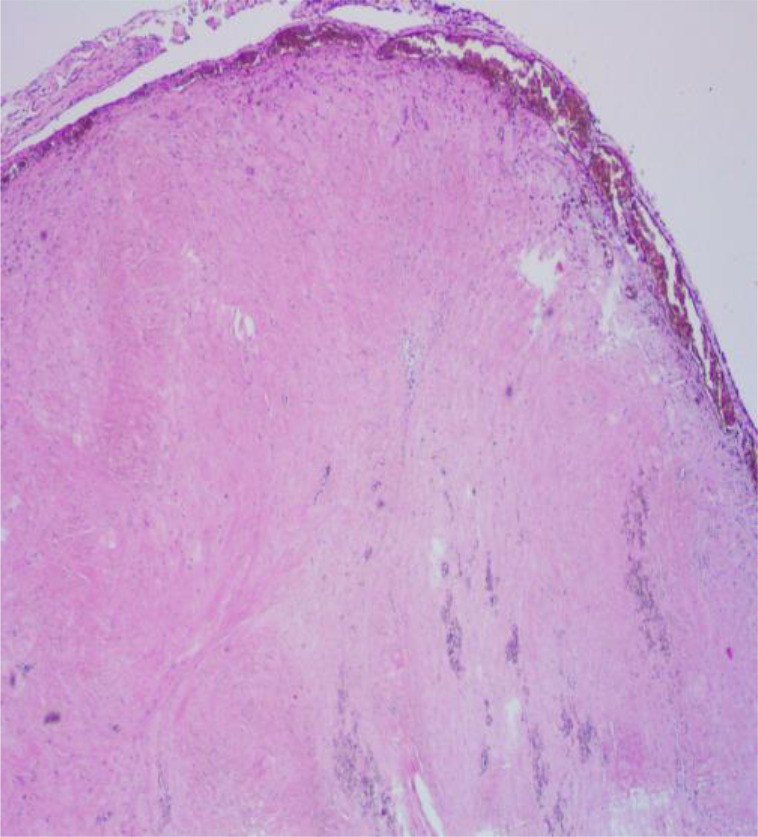
SMAT testified pathologically ×40

The relevant examinations after admission preliminarily ruled out diseases such as gastrointestinal perforation, acute pancreatitis, acute intestinal obstruction, ectopic pregnancy rupture, ovarian cyst pedicle torsion, etc. But the emergent CTA showed a filling defect in superior mesenteric artery, with no aneurysm or arterial rupture formation. (Fig. 1A,B,C) .So the patient was diagnosed with acute superior mesenteric artery thrombosis(ASMAT) and exploratory laparotomy was carried out. During operation, small intestine necrosis about 20 centimeters length was found, and no pulse was found in superior mesenteric artery. Then superior mesenteric artery incision, thrombectomy and partial resection of necrotic small intestine were performed in emergency. The patient recovered unevently and discharged after 7 days, and routine pathologic examination testified the diagnosis of SMAT (Fig. 2).

There were no reports about toremifene effect on arterial thrombosis, considering its adverse events similar to tamoxifen and reported ATSP caused by tamoxifen, we recommend a high degree of suspicion of SMAT caused by toremifene in this patient. Toremifene therapy was discontinued after the surgery, and the patient had no further thrombotic events during 1.5 years of follow-up.

## Literature review

The electronic database PubMed was searched for relevant studies, using the following terms: toremifene or fareston, tamoxifen, thrombus or embolism, thromboembolism. We found no reports about ATSP caused by toremifene, considering the similar effective mechanism of toremifene and tamoxifen, we retrieved the articles about thrombosis related with tamoxifen, especially articles about arterial embolism were analyzed.

Literature data showed no difference in the incidence rate of thromboembolic events between toremifene and tamoxifen treatment groups [4]. Such events as deep vein thrombosis (DVT), pulmonary embolism (PE) and cerebrovascular or cardiovascular thrombosis were relatively usual, but ATSP caused by toremifene is rare, with only a handful of case reports published to date. We reviewed the few reports about ATSP and analyzed these patient’s characters. Dahan et al. reported 2 cases of ATSP caused by tamoxifen during adjuvant treatment of breast cancer. The first case was a 47-year-old woman, who had no basic diseases such as blood vessels and heart, and had normal coagulation function. She suffered from thrombosis of left iliac and femoral artery during tamoxifen therapy after breast cancer surgery and chemotherapy, and died of thrombosis. The second case was a 52-year-old woman. Seven months after breast cancer operation, acute ischemia of the left lower limb was found during tamoxifen treatment. This patient also had no cardiovascular disease, and the coagulation indexes were normal. Arteriography showed thrombosis of the left femoral artery, and the embolectomy was successfully performed. The authors pointed out that these thrombosis may be related to the abnormal estrogenic effect of tamoxifen [5].

Deshmukh et al. reported a 49-year-old breast cancer patient who suffered from left tibial artery thrombosis during tamoxifen treatment. The patient had a total mastectomy 3 years earlier and received tamoxifen therapy from then on. An arteriogram showed thrombosis in her tibial arteries. After successful thrombolysis with urokinase, tamoxifen was stopped, and the blood circulation of both legs returned to normal [6]. We also collected cases of arterial thrombosis during the treatment of other diseases with tamoxifen. Richard et al. reported that a 55-year-old man developed digital artery thrombosis after using tamoxifen to treat mesenteric panniculitis for 1 week. After embolectomy, the blood flow was successfully reconstructed [7].

Considering the above 4 cases ATSP caused by tamoxifen, the mean age of ATSP onset was 52 years, and the duration of tamoxifen treatment ranged from 1 week to several years. The embolized artery included small artery (digital artery) and main artery (iliac and femoral artery), tended to location in limbs artery. Our patient, like the above cases, the age was 54 years old and took toremifene for 2 months before embolism onset. However, the embolized position in our case was in superior mesenteric artery (main visceral artery), different from limbs artery.

## Discussion 

Acute SMAT is a rare cause of acute mesenteric ischemia(AMI), with an estimated incidence of 0.09–0.2% of all acute surgical admissions [8]. Severe abdominal pain, vomiting and diarrhea, are typical symptoms of acute SMAT, which are inconsistent with the physical examination. Patient has usually no positive signs when no intestinal necrosis and peritonitis happened, which makes it difficult to distinguish SMAT from general acute abdomen, leading to misdiagnosis of SMAT. Atherosclerosis and dyslipidemia are considered as the main risk factors of SMAT [8]. Other risk factors include heart failure, atrial fibrillation, mitral valve disease, endocarditis, hypoperfusion, history of thromboembolism, etc.

It is widely recognized that cancer itself is related to increased risk of thromembolism, and chemotherapy in patients with cancer may increase this risk. Venous thromboembolism is relatively common in breast cancer patients, but arterial thrombosis, especially acute mesenteric ischemia associated with chemotherapy or endocrinotherapy rarely occurs in breast cancer patients. There were few reports about acute SMAT in cancer patients who underwent chemotherapy, but no reports of acute SMAT caused by endocrine-therapy [9, 10]. Tamoxifen and toremifene are used widely in hormone-receptor positive breast cancer patients, and their side effect include mainly gynecologic, ocular events, and thromboembolism. Thromboembolic events happened occasionally in tamoxifen or toremifene treated patients and influenced patients’ life seriously. Mata-analysis showed no difference in the incidence of deep vein thrombosis (DVT), pulmonary embolism and cardivascular or cerebrovascular events between tamoxifen and toremifene treatment groups [11]. Therefore, toremifene may be a safe alternative to tamoxifen in adjuvant endocrine therapy for breast cancer patients. Jiang et al. reported tamoxifen increases the risk of venous thromboembolism by 2–7 fold, but it is uncertain about its impact on risk of arterial thrombosis [12]. Saphner et al. reported the incidence of arterial thrombosis in premenopausal patients receiving endocrine-therapy and chemotherapy was significantly higher than that in patients receiving chemotherapy alone (1.6% vs. 0.0%, *P* = 0.004) [13]. This suggests that endocrinotherapy may increase the risk of arterial thrombosis. In our case, she underwent 4 cycles chemotherapy of TC regimen, then accepted toremifen endocrinotherapy because of positive estrogen receptor. She suffered from acute SMAT after 2 months toremifen treatment, the clinical character similar to Saphner’s report [13]. While this patient had a history of breast cancer, she was on adjuvant toremifene therapy without evidence of recurrence. She also had no prior history of thromboembolism or clotting disorders. Therefore, we consider that this case of acute SMAT may be a rare adverse event of toremifen.Arterial thrombosis is rare during toremifene treatment, and acute SMAT was not reported in literatures. In view of the high risk and rarity of acute SMAT caused by toremifene, we suggest that except for venous thrombosis, ATSP should be kept in mind during use of toremifene. Once a thrombotic event occurs, toremifene should be stopped immediately.

Of course, our study also has certain limitations. Patients with malignant tumors are already in a state of hypercoagulability, and chemotherapy may induce thrombosis. Therfore, it is uncertain that the role of toremifen in SMAT, direct or indirect? In future research, we will identify the optimal predictive factors for endocrine therapy-related ATSP to prevent its occurrence.

## Data Availability

The data sets used and/or analyzed during the current study are available from the corresponding author on reasonable request.

## Abbreviations

SMAT: Superior mesenteric artery thrombosis
TC: Paclitaxel + cyclophosphamide
ATSP: Arterial thrombosis in special position
DVT: Deep vein thrombosis
DFS: Disease free survival
OS: Overall survival
PE: Pulmonary embolism
AMI: Acute mesenteric ischemia
